# Dissociable Effects of Monetary, Liquid, and Social Incentives on Motivation and Cognitive Control

**DOI:** 10.3389/fpsyg.2020.02212

**Published:** 2020-09-09

**Authors:** Jennifer L. Crawford, Debbie M. Yee, Haijing W. Hallenbeck, Ashton Naumann, Katherine Shapiro, Renee J. Thompson, Todd S. Braver

**Affiliations:** Department of Psychological & Brain Sciences, Washington University in St. Louis, St. Louis, MO, United States

**Keywords:** social motivation, cognitive control, primary incentives, reward, decision-making

## Abstract

Humans are social creatures and, as such, can be motivated by aspects of social life (e.g., approval from others) to guide decision-making in everyday contexts. Indeed, a common view is that people may have stronger orientation toward social goals or incentives relative to other incentive modalities, such as food or money. However, current studies have only rarely addressed how social incentives compare to other types of rewards in motivating goal-directed behavior. The current study tested this claim; across two separate experiments, the effects of liquid and social incentives were compared in terms of their subsequent impact on task performance and self-reported affect and motivation. Critically, valenced social incentives offered both ecological validity (short video clips—Experiment 1) and continuity with prior stimuli used in the social reward and motivation literature (static images—Experiment 2) when examining their effect on behavior. Across both studies, the results replicate and extend prior work, demonstrating robust effects of liquid incentives on task performance and self-reported affect and motivation, while also supporting an interpretation of weaker motivational and affective effects for social incentives. These patterns of results highlight the complex and wide-ranging effects of social incentives and call into question the effectiveness of social incentives, relative to other incentive modalities, in motivating behavior.

## Introduction

Incentives are recognized as powerful sources of motivation that support the pursuit of goal-directed behavior. Although most of this evidence comes from studies using monetary incentives (e.g., Braver et al., 2014; Botvinick and Braver, 2015), recent work has begun to highlight the utility of other primary incentive types, such as social and liquid rewards, in motivating goal-directed behavior and decision-making (Krug and Braver, 2014; Tamir and Hughes, 2018). Indeed, social incentives have been shown to increase performance to the same extent as monetary rewards on a cognitive control task (Ličen et al., 2016). Social incentives have also been demonstrated to increase attentional orienting for trials associated with positive social reward (Anderson, 2016; Hayward et al., 2018) and to also increase attentional control (Ličen et al., 2019). Likewise, liquid incentives have been shown to enhance performance on challenging cognitive tasks (Beck et al., 2010; Yee et al., 2016; Yee et al., 2019). Taken together, this work provides initial evidence of the utility of diverse primary incentive modalities in guiding goal-directed behavior.

However, despite the potential utility of both social and liquid incentives in motivating goal-directed behavior and decision-making, an unanswered question is whether both primary incentive types operate equivalently in this regard. Indeed, the few studies that do examine the motivational effects of social incentives, relative to other incentive types, have reported inconsistent motivational effects. For example, both humans and non-human primates have been willing to forgo other types of rewards (e.g., money rewards for humans, juice rewards for primates) to receive social incentives, suggesting that social incentives may hold greater value compared to other types of reward (Deaner et al., 2005; Jones and Rachlin, 2006). In contrast, other recent work suggests that social, consummatory, and monetary rewards operate equivalently in motivating behavior when they are equated in terms of their subjective value (Lehner et al., 2017). Further, some evidence suggests that social incentives *do not* increase task performance to the same extent as monetary incentives, showing higher hit rates for monetary, relative to social, rewards (Rademacher et al., 2014). The heterogeneity in the effects of social incentives has been suggested to be potentially attributed to individual differences in personality (Radke et al., 2016), although there has not yet been strong support for this claim. From these findings, it is evident the literature on social incentives is quite mixed, and that there is not yet a clear understanding of the mechanisms that underlie how social incentives motivate behavior and decision-making, relative to other incentive types. Thus, an important and necessary step for clarifying these mechanisms is the development of experimental paradigms that explicitly compare and measure the effect of social and non-social incentives on task performance and goal-directed behavior.

Nevertheless, evaluating the motivational impact of social incentives solely in terms of task performance (i.e., objective measures) may neglect other important signatures, such as influences on affective/emotional reactions and other metrics of subjective experience. Indeed, there is some evidence to suggest that affect and motivation explain unique variance in modulating cognitive control (e.g., Dreisbach and Goschke, 2004; Chiew and Braver, 2011). In particular, some theoretical accounts postulate that reward incentives could impact dissociable neural pathways related to the hedonic/affective (“liking”) and motivational (“wanting”) dimensions activated by such incentives (Berridge and Robinson, 2003). Further, other theoretical frameworks highlight the unique contribution affective processes have on decision-making and goal-directed behavior (Winkielman et al., 2007). Based on such accounts, it is plausible that social incentives could elicit affective responses that operate distinctly from their motivational impact on cognitive task performance, which could lead to differential patterns of performance across primary incentive types. For example, it is possible that social incentives might have a similar impact on cognitive task performance compared to primary incentives, such as liquids, but that social incentives would elicit stronger affective and other subjective responses. Likewise, given the increased evolutionary importance of social cognition and social motivation for humans (i.e., Pyszczynski et al., 1997), it is plausible that social incentives could exert a stronger influence over cognitive processing and behavior than other primary incentives, such as liquid.

The current study aimed to test this question by adapting an incentive integration paradigm developed in our lab (Yee et al., 2016, 2019) to investigate how participants integrate the motivational value of monetary incentives with liquid delivery used as performance feedback. This paradigm is an innovative one in that it has demonstrated utility for examining the combined effects of monetary and non-monetary incentives on cognitive task performance, especially for incentives varying across motivational valence (e.g., positive/approach vs. negative/avoidance). The key innovative aspect of the paradigm to highlight is its ability to isolate and quantify the effects of primary (e.g., liquid) incentives on cognitive task performance, separately from the effects of secondary (i.e., monetary) incentives. In the paradigm, monetary reward incentives are offered to participants for fast and accurate performance, with the value of the incentive manipulated on a trial-by-trial basis (i.e., low, medium, or high value). Successful attainment of the monetary reward is signaled to participants via post-trial feedback (e.g., in liquid incentive conditions, oral-delivery of liquid into the participant’s mouth). Critically, the meaning of the incentive feedback is purely symbolic (i.e., a drop of liquid signals successful attainment of monetary reward under all conditions regardless of its valence), which makes it possible to examine motivational influences of non-monetary incentives in terms of their incidental, or obligatory, impact on performance. Importantly, the use of monetary rewards as the explicit incentive offered and manipulated across trials biases participants to an approach-motivated state (Bijleveld et al., 2012), which is reflected in overall high-performance levels. Thus, it is possible to quantify the effects of motivational valence of the post-trial feedback on task performance. Indeed, in several prior studies using this incentive integration paradigm with liquid incentives, we found evidence to support that performance is enhanced on positively-valenced (liquid) trials relative to the neutral-valence, and impaired on negatively-valenced trials (relative to positive and neutral; Yee and Braver, 2018).

In the current study, we extended the paradigm to additionally examine the effects of social incentives on task performance (compared to liquid incentive effects) in two parallel and complementary experiments. In particular, we utilized a within-subjects design to enable direct comparison of the two incentive types. In Experiment 1, the social incentives were a novel set of dynamic stimuli (i.e., short video clips) that provided motivationally valenced feedback (positive, neutral, or negative; Tully et al., 2017). These stimuli were chosen to provide an ecologically valid type of social message that participants might experience in daily life when faced with decision-making prospects (e.g., compliments, insults). Ecological validity is a critical consideration when considering extant research on social incentives, since there have been concerns raised as to whether the social content of stimuli being used to investigate social motivation in prior studies might be too simplified and decontextualized to approximate the social feedback individuals experience in daily life (i.e., Tamir and Hughes, 2018). In Experiment 2, the objective was to provide greater continuity with the prior literature, by using static images of valenced facial expressions as social incentives. As these static images form the basis of much of the extant research on social incentives (e.g., Cloutier et al., 2008; Spreckelmeyer et al., 2009), this approach offers a more clear-cut extension and comparison with these prior bodies of work, despite lacking the ecological validity of the stimuli used in Experiment 1. Importantly, we believed that the inclusion of both types of social incentives (static facial expressions, dynamic social messages) would provide a fuller picture of when and how social incentives may be effective in terms of their influence on motivated behavior and affect. Across both studies, we predicted that participants would not only be able to integrate liquid with monetary incentives, as we found in our prior studies (Yee et al., 2016, 2019), but would also show integration effects with social and monetary incentives, providing further evidence of the utility of using both primary incentive types in motivating goal-directed behavior.

Further, we assessed participants’ self-reported affect in both liquid and social feedback conditions in order to provide initial information regarding the relationship between affective and motivational dimensions of the feedback. These ratings were only collected in Experiment 1, as data collection was already underway in Experiment 2 when we implemented this portion of the experimental protocol. We predicted that affective modulation would be stronger in the social feedback condition relative to liquid feedback, consistent with a potential dissociation between the role of affect and motivation in modulating cognitive control (e.g., Chiew and Braver, 2011). Obtaining this pattern of results would provide further evidence for the distinct roles of affect and motivation in the recruitment of cognitive control. Conversely, an alternative outcome would be if the liquid feedback condition elicited both a stronger motivational influence on performance and also had a stronger impact on self-reported affect. Such an outcome would indicate that the affective and/or motivational influences of liquid feedback are greater than those for social feedback. To preview, the results from both studies appear to provide greater support for this latter, alternative interpretation, reinforcing the utilization of liquid feedback as an effective motivational incentive for modulating cognitive task performance.

## Experiment 1

In this study, we directly compared the motivational influences of social and liquid incentives when each was used as performance feedback. To investigate this question, we utilized an incentive integration paradigm originally developed by Yee et al. (2016). The key feature of this paradigm is that it provides a means of determining whether valenced performance feedback—positive, neutral, negative—is integrated with pre-trial monetary incentive cues to modulate the motivational value onto task performance. In the original studies (Yee et al., 2016, 2019), liquid incentives were used as feedback, with liquid delivery indicating to the participant that they had successfully obtained the monetary reward available on that trial (through fast and accurate responding). Because the motivational valence of the liquid was manipulated across blocks (positive, neutral, or negative in different blocks), it was possible to detect the additive effect of the liquid on task performance, since performance was better on positive blocks and worse on negative blocks, relative to neutral.

Here, the paradigm was adapted to also compare the effects of liquid and social incentives. In the social incentive condition, short video clips were substituted as performance feedback instead of liquid delivery. Presentation of a video to participants as post-trial feedback had the same meaning in this condition, always indicating success at attaining the monetary reward available on that trial. Yet again, the motivational valence of the video clip was manipulated, such that positive, neutral, or negative messages were delivered in different blocks. This provided the ability to test whether the valence of the video message had a unique impact on task performance. Moreover, by implementing a within-subjects design, it was possible to directly compare social incentive effects with the effects of liquid incentives, as all participants performed both incentive conditions in different experimental sessions.

### Materials and Methods

#### Participants

Forty-one participants (29 females; ages 18–37 years; *M* = 20.71, *SD* = 3.26) were recruited from Washington University Psychology Department and Washington University School of Medicine Volunteers for Health subject pools. Participants completed two separate sessions at Washington University in St. Louis, at least 24 h apart. All participants provided written informed consent and were given payment of $10/h in addition to task-based earnings contingent upon fast and accurate performance in the incentive blocks. Ten participants were excluded from analysis; four only completed one session and did not return for the second, three ended a session prior to completion, one failed to comply with task instructions during the first session and was not invited back to complete the second session, and two participants’ data were unable to be analyzed due to experimenter error during data acquisition. Consequently, the final sample subjected to analysis consisted of 31 participants (21 females; ages 18–37 years *M* = 20.81, *SD* = 3.41). All participants were native English speakers, reported no current or previous history of neurological trauma, seizures, or mental illness, and no use of psychotropic medications. The Washington University Human Research Protections Office approved all experimental procedures.

A power analysis was performed using G^∗^Power (Faul et al., 2007) to identify the sample size necessary to detect effects of both monetary and liquid incentives, at the same level of effect size observed in the original work with this paradigm (Yee et al., 2016). The analysis revealed that 28 participants would be necessary for 80% power to detect such effects.

#### Tasks

All participants performed an incentivized cued-task switching paradigm following the same basic structure as Yee et al. (2016, 2019). The task-switching paradigm was administered using E-Prime Version 2.0.10.242 (Psychology Software Tools, Pittsburgh, PA, United States) and consisted of a series of trials in which participants randomly alternated between performing letter and digit categorization tasks, with the task to be performed on each trial indicated by an advance task cue. Task responses were recorded using an E-prime stimulus response box, via button presses made with their right index and middle fingers, according to response mappings that were counterbalanced across participants. Each trial began with a fixation cross, which was displayed for 300 ms. Next, a task cue was presented for 500 ms, which indicated the categorization task to be performed on that trial. If the cue was “Attend Letter” the participant needed to categorize the letter as being either a vowel or a consonant, whereas the “Attend Number” cue indicated that the participant needed to categorize the number as being either odd or even.

In the primary task conditions, trials were incentivized through monetary rewards available on each trial and indicated through advance reward cues accompanying the task cues. Specifically, monetary reward cues appeared above and below the “Attend Number” or the “Attend Letter” cues ($ = low reward, $$ = medium reward, or $$$$ = high reward). The number of dollar signs varied from trial to trial. During incentive trials, participants were informed that the dollar signs represented the relative monetary worth of that trial (e.g., $$ trials being worth twice as much as $ trials and half as much as $$$$ trials). As described further below, these monetary rewards could be obtained through fast and accurate performance. Thus, participants were incentivized to maximize monetary reward earnings, which occurred by enhancing cognitive task performance (e.g., faster and accurate responses).

Prior to the primary task conditions, participants also performed the task under practice and baseline conditions, in which no rewards were available. The same dollar sign cues were presented, however, but during these trials, participants were told that dollar signs held no significance. Following the task (+monetary reward) cue, a blank screen was presented for 1850 ms, followed by the target stimulus which was presented for up to 2000 ms. The target stimulus was ambiguous as to the relevant tasks, since it always consisted of both a letter and a number, and the same two response buttons were used in each task (e.g., one response mapping might be to respond middle finger for odd, index finger for even in the Digit task, and middle finger for vowel and index finger for consonant in the Letter task). As such, the task placed high demands on cognitive control, requiring participants to mentally update the appropriate task goal and associated response rules on a trial-by-trial basis in order to perform successfully. The baseline condition was used to set an RT criterion for later incentivized trials, as described below.

**FIGURE 1:**
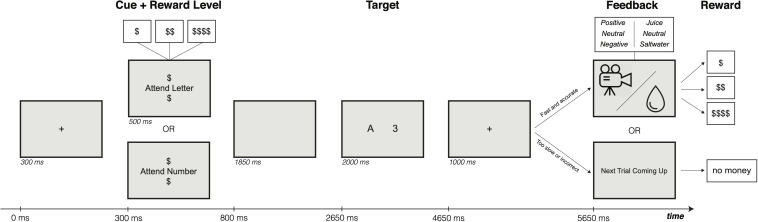
Incentive integration task paradigm. Participants were asked to perform a consonant-vowel odd-even (CVOE) switching task. Reward cues indicated the relative amount of monetary reward available on each trial given fast and accurate performance. If participants were accurate and faster than a subject criterion response time (30% of fastest response times for correct trials during the baseline block), then they received feedback (social or liquid) at the end of the trial. If subjects answered incorrectly, too slowly, or not at all, they received neither monetary reward nor liquid/social incentives.

The key feature of the task was the social or liquid feedback delivered to participants following their response during the primary task conditions. This feedback was delivered only when participants were both accurate and fast on the trial (with the response time cutoff set individually according to baseline task performance; see below). Thus, feedback symbolically indicated to participants that they were successful in obtaining the available monetary reward on that trial (high, medium, or low). However, in different blocks the motivational valence of the feedback was manipulated (positive/appetitive, neutral/neutral, or negative/saltwater). Given that the symbolic meaning of the feedback was positive and held constant across blocks, any further influence of the feedback on performance can be taken as an indicator that it had unique motivational value, over and above the value of the monetary incentive. Indeed, in prior work using liquid feedback (Yee et al., 2016, 2019), there were robust effects of feedback valence on task performance, in that participants received reward feedback at a higher rate when the liquid was of positive/appetitive valence (apple juice) and at a lower rate when the liquid was of negative/aversive valence (saltwater) relative to neutral (tasteless solution). On trials in which the participant made an error or was too slow, they instead received a visual message that read “Next Trial Coming Up”; see Figure 1 for a schematic diagram. During practice and baseline conditions (when no incentives were available), participants instead received only visual feedback; on practice trials, participants received feedback indicating whether they were correct or incorrect, or did not respond within the available response window. Baseline trials did not provide participants with performance feedback, they instead received a visual message that read “Next Trial Coming Up,” regardless of task performance.

#### Procedure

Before the start of the experimental session, the neutral and saltwater liquid solution were prepared in a testing room in the lab. The isotonic neutral solution consisted of 1 liter of distilled water, 0.0495 g of NaHCO3 (Sodium Bicarbonate), and 0.4668g of KCl (Potassium Chloride) diluted to 25% for use in the study. The saltwater solution consisted of 250 mL of distilled water and 2.1915 g of non-iodized salt. The juice used was 100% apple juice (Mott’s brand) and purchased from the store. To minimize the likelihood that participants were already satiated during the experimental sessions, they were asked to abstain from eating or drinking anything besides water for two hours prior to the session. Upon arrival, participants used REDCap, a secure research and experience management software program (Harris et al., 2009), to complete a contact information questionnaire and pre-task self-report individual difference questionnaires that were not the primary focus of analyses (see Supplementary Material).

Each participant performed two experimental sessions that were identical in structure and only differed in the incentive condition being performed (social feedback, liquid feedback). Session order was counterbalanced across participants. Each session began with a practice and baseline phase in which the cued-task switching paradigm was performed under non-incentive conditions. The practice phase consisted of two blocks in which participants practiced only a single task, either the letter or number categorization task. One cue, either “Attend Number” or “Attend Letter,” was presented for all trials of the block (12 trials per task, counterbalanced order). A third practice block consisted of both number and letter trials, intermixed (24 trials total). After the practice phase, participants performed a baseline phase, which consisted of three longer blocks mirroring the structure of the practice blocks. During the baseline blocks, participants performed the same tasks as in the practice blocks, and counterbalanced in the same order, but received no feedback after each trial. The first two baseline blocks (either single-task letter or number) consisted of 48 trials each and the third task-switching block (intermixed number and letter trials) consisted of 96 trials. In each of the baseline runs, participants were instructed to perform as quickly and accurately as possible.

Performance on the baseline task-switching block was used to compute the reward cutoff time in subsequent incentive blocks. Specifically, the reward criterion was calculated individually for each participant, based on the 30th percentile of their correct reaction times in the mixed baseline run performed during that session. On incentive trials, participants had to be both accurate and faster than this cutoff time to receive the monetary reward available on that trial. Prior to beginning the incentive blocks, participants were given this information regarding the criteria for being rewarded, and were told that they had the opportunity to earn an additional $14 across the two experimental sessions, in addition to their hourly pay.

The liquid feedback session matched identically the structure used in prior work (Yee et al., 2016, 2019) and consisted of six incentive blocks total, two consecutive blocks each performed with three different liquids associated with a different affect/motivational valence: positive (apple juice), neutral (isotonic tasteless solution), negative (saltwater). The block order of the three liquids was counterbalanced between participants. Each block consisted of 48 trials, and participants were given a rest break between each block. Liquid was dispensed (2 mL per trial) using a digital infusion pump (model SP210iw, World Precision Instruments Inc., Sarasota, FL, United States) with Tygon tubing (United States Plastics Corporation, Lima, OH, United States) delivering liquid directly into the participant’s mouth. As described above, on all blocks, participants only received the liquid available for that block on trials in which they were both accurate and their response latency was faster than their individually determined reward cutoff time. Consequently, although the symbolic meaning of the feedback was the same in all cases (indicating success at obtaining the monetary reward), the motivational meaning varied, and was predicted to combine with the monetary reward value in an additive manner, positively in the juice blocks, and negatively in the saltwater blocks, relative to the neutral blocks.

The social feedback session involved a parallel structure, also consisting of six blocks, in sets of two blocks of 48 trials each, performed consecutively, with a rest break provided after each block. Each set was associated with social feedback of a different affect/motivational valence (positive, neutral, and negative), with valence order counterbalanced across participants. The social feedback was presented in the form of a short video clip, selected from the SocialVidStim set (Tully et al., 2017). The SocialVidStim is a collection of short video clips (*N* = 4,673), each approximately six seconds in length, featuring a single male or female actor facing directly to the screen (i.e., as if speaking to the participant), and delivering an affectively valenced message. Examples of social feedback presented to the participants are as follows (for videos and further information)^1^ : “People think positively of you” (positive); “You are a disappointment” (negative); “A minute is a unit of time” (neutral). In brief, validity data collected to date on a subset of videos (*N* = 1,001; 428 negative; 429 neutral, 144 positive) from 1,781 participants indicate that negative videos are perceived as more negatively valenced (mean difference = −1.21; 95% CI [−1.23, −1.19]) and more arousing (mean difference = 0.68, 95% CI [0.66,0.70]) than neutral videos. Similarly, positive videos are perceived as more positively valenced (mean difference = 0.85, 95% CI [0.82,0.88]) and more arousing (mean difference = 0.59, 95% CI [0.55,0.63]) than neutral videoas. Test-retest reliability data collected on a subset of videos (*N* = 232; 86 negative, 92 neutral, 54 positive) from 354 participants indicate good-to-excellent reliability of negative and positive videos (ICC negative = 0.93; ICC positive = 0.87) and moderate reliability of neutral videos (ICC neutral = 0.66). For this experiment, we selected 165 videos (55 of each valence type) from the SocialVidStim, which featured 26 different actors (13 females; ages 18–41, *M* = 25.0, *SD* = 5.2; 4 Asian, 2 Black, 19 Caucasian, 1 more than one race).

As in the liquid feedback condition, social feedback messages were all of the same category for a given block and were only received on trials in which the participant was both accurate and faster than their individually determined reward cutoff time. Therefore, paralleling the liquid condition, the symbolic meaning of the social feedback was the same in all cases (indicating success at obtaining the monetary reward), the affective/motivational meaning varied, and was predicted to combine with the monetary reward value in an additive manner, either positively or negatively, relative to neutral as a function of the block condition.

Following each incentive run, participants completed ratings of their current affective state, using a 5-point scale. Participants were instructed to indicate “to what extent you feel this way right now” for each of the 10 valenced words; three terms coded negative affect valence (Ashamed, Irritable, Upset), three coded positive affect valence (Inspired, Content, Excited), and four coded arousal (Fatigued, Alert, Determined, Stressed). The ratings for valence terms were averaged together to create composite positive and negative affect scores in the primary analysis. Additionally, after completing all task blocks, post-task questionnaires were given in each session that assessed ratings of how much participants liked the incentive type (e.g., social or liquid feedback), how intense or arousing they found the stimuli, and also self-reported levels of motivation, liking, and performance for each incentive type (e.g., $-positive social feedback, $$$$-saltwater) using a seven-point Likert scale. A complete description of all self-report questionnaires is provided in the Supplementary Material. All relevant experimental scripts, data, and analyses are located in an online repository on the Open Science Framework: https://osf.io/pu9gs/.

#### Data Analysis

Across both experiments, the primary analysis approach consisted of a 3-factor, within-subject ANOVA [3 monetary reward levels (low, medium, high) × 3 feedback valences (positive, neutral, negative) × 2 incentive types (liquid, social)] in order to test for the effects of the task conditions on the primary dependent measure, reward rate (i.e., the subjective motivation to engage in cognitive control), followed by *post hoc* tests when interactions were identified. In Experiment 1, we also used a 2-factor, within-subject ANOVA design to examine the effects of incentive type (liquid, social) and feedback valence (positive, neutral, negative) on participants’ self-reported affect after performing each task block. As such, we present the results from each ANOVA using the test F-statistic, significance level of the effect, and the effect size estimate. The effect sizes for the ANOVAs are reported using the generalized eta squared metric, which is the preferred method for reporting effect sizes of within-subject ANOVA designs (Lakens, 2013). Further, when pairwise comparisons were made using t-tests, we report the results using the t-test statistic, significance level of the effect, and the effect size estimate (Cohen’s *d*). No data were excluded, on the basis of outliers, in either study.

### Results

#### Task Performance

We used reward rate (i.e., the percentage of rewarded trials in each incentive condition) to quantify each participant’s subjective motivation to implement cognitive control to earn the incentives offered both in the liquid and social feedback tasks. Because the expected reward rate was 0.3, assuming no change in motivation from the baseline condition, the first analysis tested whether the average reward rate obtained in each condition exceeded this value. Indeed, across both the liquid and social feedback sessions, participants exceeded this value (liquid: 31/31, social: 30/31), suggesting that cognitive control was enhanced when participants performed cued task-switching under the heightened motivational context associated with incentives.

The primary analysis was a full 3-factor ANOVA enabling comparisons between all of the task conditions (3 monetary reward levels × 3 feedback valences × 2 incentive types). In this analysis, there was a main effect of monetary reward, *F*(2,60) = 18.53, *p* < 0.001, $\eta_{\text{G}}^{2}$ = 0.024, indicating that reward rate was highest on trials with the highest monetary rewards available (*M* = 0.679, *SD* = 0.092), and was lower when both medium (*M* = 0.626, *SD* = 0.099) and small rewards (*M* = 0.632, *SD* = 0.096) were available. The monetary reward level did not further interact with incentive session, *F*(2,60) = 2.13, *p* = 0.127, $\eta_{\text{G}}^{2}$ = 002. Further, there was no effect of incentive type on task performance, *F*(1,30) = 0.99, *p* = 0.327, $\eta_{\text{G}}^{2}$ = 0.009. This suggests that, as expected, participant performance was sensitive to the monetary rewards and did not qualitatively differ across social and liquid feedback.

**FIGURE 2:**
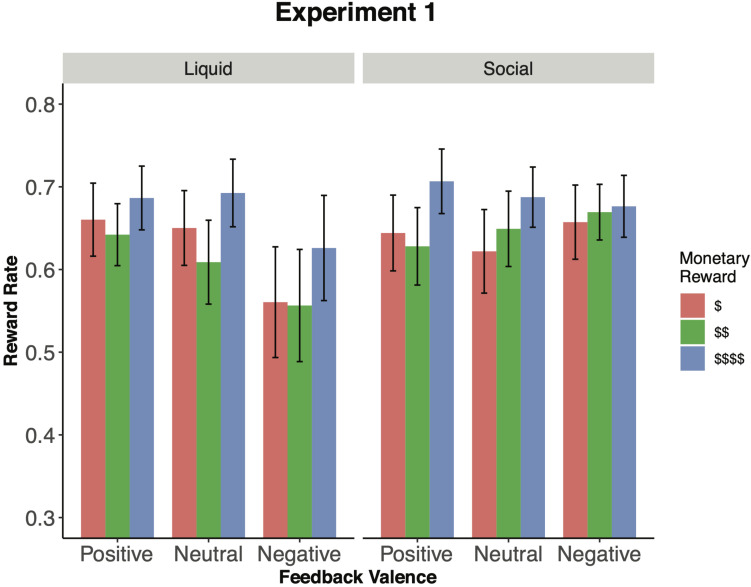
Incentive integration effects by experimental manipulation (Experiment 1). The figure shows reward rate by monetary reward level and feedback (i.e., liquid, social). Participants showed a main effect of monetary reward and feedback during the liquid feedback session. However, there was only a main effect of monetary reward, but not feedback during the social feedback session. Error bars represent 95% confidence intervals around the mean.

We next focused on the effects of feedback valence. Although there was no main effect *F*(2,60) = 2.82, *p* = 0.067, $\eta_{\text{G}}^{2}$ = 0.010, feedback valence did interact with incentive type, *F*(2,60) = 5.56, *p* = 0.006, $\eta_{\text{G}}^{2}$ = 0.018, suggesting that valence effects were distinct for the social feedback relative to the liquid feedback. However, feedback valence did not further interact with monetary reward, *F*(4,120) = 0.53, *p* = 0.718, $\eta_{\text{G}}^{2}$ = 0.001, nor was the 3-way interaction significant, *F*(4,120) = 2.28, *p* = 0.065, $\eta_{\text{G}}^{2}$ = 0.005. To decompose the feedback valence × incentive type interaction, we examined the feedback valence effect in each incentive type (i.e., social, liquid) separately. In the liquid feedback, we observed the expected effect of feedback valence, *F*(2,60) = 5.16, *p* = 0.009, $\eta_{\text{G}}^{2}$ = 0.027, whereby juice trials had higher reward rate (*M* = 0.663, *SD* = 0.100) than saltwater trials [*M* = 0.581, *SD* = 0.208; *t*(30) = 2.46, *p* = 0.020, *d* = 0.462; Figure 2]. This pattern replicates the findings of the prior liquid feedback studies (Yee et al., 2016, 2019) and suggests that participants were able to additively combine liquid and monetary rewards to modulate task performance. In contrast, the social feedback condition did not produce any evidence of reliable integration effects, as feedback valence showed no influence on reward rate (*F*[2,60] = 0.51, *p* = 0.601, $\eta_{\text{G}}^{2}$ = 0.002). These conclusions were supported by supplemental analyses examining the effects of incentive session on reaction time and accuracy data (see Supplementary Material for comprehensive descriptive summaries and analyses).

#### Affect Ratings

**FIGURE 3:**
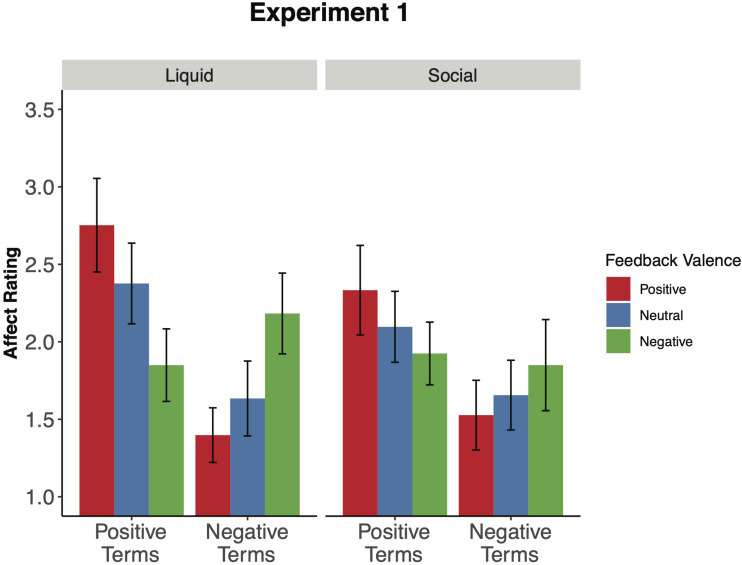
Affect ratings by experimental manipulation (Experiment 1). This figure illustrates the mean affect rating for each incentive condition across both liquid and social feedback sessions. Participants rated the extent to which they were feeling each of the emotion words after each block of the cued task-switching paradigm using a five-point scale (e.g., 1-*not at all*, 5-*extremely*). Positive affect represents the average ratings across the following terms: “content,” “inspired,” and “excited.” Negative affect represents the average ratings across the following terms: “ashamed,” “irritable,” and “upset.” Error bars signify 95% confidence intervals around the mean.

One question is whether there might be a dissociation between the motivational and affective impact of social and liquid incentives. As a means of addressing this issue, we examined the positive and negative affect ratings that participants self-reported following each incentive block for both the liquid and social incentives. Overall, there were no differences in affect ratings across the two incentive types, *F*(1,30) = 2.40, *p* = 0.132, $\eta_{\text{G}}^{2}$ = 0.007; see Supplementary Material for a complete results on all ratings. Likewise, the effect of incentive type did not interact with feedback valence, *F*(2,60) = 0.017, *p* = 0.983, $\eta_{\text{G}}^{2}$ < 0.001, or affect term (positive, negative), *F*(1,30) = 1.10, *p* = 0.303, $\eta_{\text{G}}^{2}$ = 0.002. Consequently, we examined each incentive type separately, to determine the degree to which each type of feedback influenced affect ratings. In the liquid feedback condition, a feedback valence × affective term interaction was observed, *F*(2,60) = 15.09, *p* < 0.001, $\eta_{\text{G}}^{2}$ = 0.122 (Figure 3). This was due to significantly higher ratings for the positive affect terms in the juice condition (*M* = 2.75, *SD* = 0.91) relative to saltwater (*M* = 1.85, *SD* = 0.72; *t*[30] = 5.67, *p* < 0.001, *d* = 1.087). Conversely, the opposite pattern was observed for the negative affect terms, such that participants rated higher levels of negative affect for saltwater (*M* = 2.18, *SD* = 0.82) relative to juice [*M* = 1.40, *SD* = 0.62; *t*(30) = 5.37, *p* < 0.001, *d* = 1.065].

Interestingly, a similar pattern was also observed in the social feedback condition, with a significant feedback valence × affective term interaction, *F*(2,60) = 4.21, *p* = 0.020, $\eta_{\text{G}}^{2}$ = 0.032. There were higher ratings for the positive affect terms in the positive social feedback condition (*M* = 2.33, *SD* = 0.96) relative to the negative social feedback condition [*M* = 1.92, *SD* = 0.73, *t*(30) = 2.45, *p* = 0.020, *d* = 0.471]. Conversely, participants rated higher levels of negative affect for the negative social feedback condition (*M* = 1.85, *SD* = 0.94) relative to the positive social feedback condition [*M* = 1.53, *SD* = 0.73, *t*(30) = 2.19, *p* = 0.036, *d* = 0.377]. This finding suggests that the social feedback condition was successful in modulating participants’ self-reported affect in alignment with the type of feedback valence received. On the other hand, the effects of feedback valence on affect were surprisingly weaker in the social feedback condition than in the liquid condition (i.e., the effect size (generalized eta-squared) of the feedback valence × affect term interaction was 0.122 in the liquid feedback session and 0.032 in the social feedback session). Thus, the affect ratings are somewhat consistent with the task performance data in suggesting a stronger affective/motivational influence of liquid feedback relative to social feedback.

### Discussion

In this experiment, we directly compared the motivational impact of social and liquid incentives when each modality was used as performance feedback in an incentive integration paradigm. Critically, this paradigm enabled us to determine whether valenced performance feedback (positive, negative, neutral) and monetary incentive cues were integrated together across both feedback modalities (social, liquid) to modulate motivation levels engaged to perform a highly challenging cognitive task. Replicating prior findings (Yee et al., 2016, 2019), in the liquid feedback condition we found effects of both monetary and liquid incentives on reward rate, suggesting that participants are able to additively combine the motivational value of these incentives to modulate task performance. In addition, for this condition, the post-block affect ratings showed robust effects of liquid feedback such that positively valenced words showed the highest ratings for juice relative to neutral, relative to saltwater (i.e., juice > neutral > saltwater), whereas negatively valenced words showed the opposite pattern (saltwater > neutral > juice), demonstrating that the liquid feedback manipulation was having a translatable effect to the participant’s current affective state. Taken together, these results provide strong confirmation of prior work (Yee et al., 2016, 2019), demonstrating that monetary and liquid incentives can combine to modulate cognitive task performance through changes in experimentally-induced motivational states. Indeed, the affect ratings extend prior work by suggesting that not only does liquid feedback alter participants’ motivational states during task performance, but that it also impacts their emotional state as well, at least to the degree that the self-reported affect terms are valid indicators of this state.

Conversely, social feedback does not appear to operate as strongly in this manner, or have a clear influence on cognitive task performance. Despite producing a numerically higher reward rate overall, we found no observable effect of social feedback on reward rate. Further, we found that the effects of social feedback on the post-block affect ratings were weaker than that observed in the liquid condition, though they followed the same pattern, with the highest ratings for the positively valenced affect terms coming in the positive social feedback condition (positive > negative), whereas the negatively valenced terms showed the opposite pattern (greatest in the negative social feedback condition). On the other hand, even though the effects of social feedback were weaker than liquid feedback, this observed pattern of results serves to validate that the manipulation was at least partially successful in having an impact on participants’ psychological states (again under the assumption that the self-reported affect ratings are valid indicators of such states). Moreover, these results also suggest that there could be a potential dissociation between affect and motivation, such that social incentives do induce changes in participants’ current affective state, but have no influence on their motivation to perform the task. Indeed, analyses of participants’ self-reported ratings of motivation were consistent with this potential dissociation (see Supplementary Material), in showing that there were no effects of social feedback valence on motivation despite participants’ self-reported changes in affect across social feedback conditions. Nevertheless, it is also possible that impact of social feedback on both affect and motivation were reliably weaker than liquid feedback conditions, but this claim needs be investigated through additional studies. To provide convergent support, a second experiment was conducted comparing the effects of liquid and social feedback, with the same incentive integration paradigm.

## Experiment 2

Experiment 1 demonstrated that primary liquid incentives are able to robustly modulate performance on a challenging cognitive task, whereas social incentives do not appear to have a translatable effect on participant motivation, as indexed by task performance. However, given the novelty of the social stimuli used in Experiment 1 (i.e., dynamic social messages), relative to the majority of the extant research on social incentives (i.e., static faces, written messages), it is unclear whether similar results would be obtained by using a more traditional means of operationalizing social incentives. To address this question, and increase continuity with the prior literature, in Experiment 2 static facial expressions were used as social feedback within the same incentive integration cued task-switching paradigm. Further, the limited sample size of Experiment 1 might have reduced our ability to detect the effects of social feedback on task performance, if such effects are not as strong as those previously observed effect for liquid feedback (Yee et al., 2016, 2019). Thus, Experiment 2 consisted of a considerably larger sample size, which enhanced our ability to detect the potentially smaller effect of social feedback on motivation. Consistent with the results of Experiment 1, we predicted that participants would be able to integrate liquid with monetary incentives, but not social and monetary, demonstrating a dissociation of primary incentive type in inducing motivational effects, and highlighting the complex and heterogenous effects of social incentives in motivating goal-directed behavior.

### Materials and Methods

#### Participants

Eighty-three participants (62 females; 18–40 years; *M* = 28.35; *SD* = 6.87) were recruited from Washington University Psychology Department and Washington University School of Medicine Volunteers for Health subject pools. All participants provided written informed consent. Participants were given payment of $10/h in addition to task-based earnings contingent upon fast and accurate performance in the incentive blocks. Although the intent was to run this study only with monetary compensation, a small subset of participants were provided with course participation credit for their time, rather than the $10/h payment schedule. Nine participants were excluded: five participants for technical errors and four participants for failure to comply with the task instructions. The final sample consisted of 74 participants (56 females; ages 18–40, *M* = 28.27, *SD* = 6.90). All participants were native English speakers and reported no current or previous history of neurological trauma or seizures. The Washington University Human Research Protections Office approved all experimental procedures.

The effect sizes of monetary and liquid rewards in Experiment 1 were smaller than what was observed in prior work with this paradigm (Yee et al., 2016). However, using the effect sizes from Experiment 1, results from a power analysis suggest that a minimum of 65 participants would be necessary for 80% power to detect such effects, motivating a larger sample size in Experiment 2.

#### Task

Experiment 2 used the same incentivized task-switching paradigm as in Experiment 1; however, rather than using dynamic social feedback messages, participants were presented with static images of faces as valenced social feedback (positive-happy, neutral-neutral, negative-sad). The face images used as social feedback were derived from a subset of the NimStim database of multiracial facial expressions (Tottenham et al., 2009). This open source stimuli set was validated for the ability of untrained participants to reliably identify the emotion (i.e., happy, neutral, sad) of each face image^2^. The overall proportion correct was robust (*M* = 0.81; *SD* = 0.19) and demonstrated high agreement across stimuli between the rater labels and intended expressions (mean κκ = 0.79; *SD* = 0.17). We utilized 202 closed- and open-mouth faces out of the 672 images contained within the set, categorizing them into valences of happy, neutral, and sad. The average validity ratings for the happy, neutral, and sad faces in the NimStim set were 0.92, 0.86, and 0.72, respectively.

#### Procedure

The experimental session was identical in structure to the procedure described in Experiment 1, however, rather than completing each incentive condition (social feedback, liquid feedback) across two separate sessions, incentive type was counterbalanced across participants within the same session. Upon completing all task blocks, participants completed post-task questionnaires on which they rated how much they liked the incentive type (e.g., social or liquid feedback), how intense or arousing they found the stimuli; they also rated their overall levels of motivation, liking, and performance for each incentive type (e.g., $-positive social feedback, $$$$-saltwater) using a seven-point Likert scale. A complete analysis of this rating data is contained within the Supplementary Material.

#### Data Analysis

The data analysis approach was identical to that described in Experiment 1, utilizing repeated-measures ANOVA (followed by *post hoc* tests where appropriate), and reporting of effect-size with the generalized eta-squared measure (or Cohen’s *d* for *t*-tests).

### Results

#### Task Performance

As in Experiment 1, we used reward rate to quantify each participant’s subjective motivation to implement cognitive control to earn the incentives offered in both the liquid and social feedback conditions. Across both incentive modalities, participants exceeded the expected reward rate (liquid: 73/74, social: 74/74), as determined by the criterion response time, suggesting that cognitive control was enhanced when participants were provided with increased motivational incentives during the task (i.e., the ability to receive rewards based on both fast and accurate task performance). However, when directly comparing the liquid and social feedback conditions, average reward rate was higher overall for social (*M* = 0.718, *SD* = 0.103) relative to liquid feedback [*M* = 0.631, *SD* = 0.130), *t*(73) = 6.75, *p* < 0.001, *d* = 0.73].

Consistent with the analyses conducted in Experiment 1, we used a full 3-factor ANOVA to provide comparisons between all of the task conditions (3 monetary reward levels × 3 feedback valences × 2 incentive types). In addition to the main effect of incentive type described above, there was also a main effect of monetary reward, *F*(2,146) = 34.714, *p* < 0.001, $\eta_{\text{G}}^{2}$ = 0.028, indicating that reward rate was highest on trials with the highest monetary rewards available (*M* = 0.721, *SD* = 0.109), and was lower when medium (*M* = 0.660, *SD* = 0.104) and small rewards (*M* = 0.651, *SD* = 0.118), *ps* < 0.001, were available. Further, monetary reward level interacted with incentive type, *F*(2,146) = 5.185, *p* = 0.007, $\eta_{\text{G}}^{2}$ = 0.004, suggesting that participant performance qualitatively differed across social and liquid feedback conditions. To decompose this interaction, we examined the effect of monetary reward for each incentive type separately. There was an effect of monetary reward for the liquid feedback condition, *F*(2,146) = 22.53, *p* < 0.001, $\eta_{\text{G}}^{2}$ = 0.036, whereby reward rate was highest for high monetary reward trials (*M* = 0.680, *SD* = 0.126), relative to medium monetary reward trials (*M* = 0.618, *SD* = 0.137), and medium monetary reward trials relative to low monetary reward trials (*M* = 0.594, *SD* = 0.169), *ps* ≤ 0.035 (i.e., $ < $$ < $$$$). Likewise, there was also an effect of monetary reward for the social feedback condition, *F*(2,146) = 16.20, *p* < 0.001, $\eta_{\text{G}}^{2}$ = 0.022, such that reward rate was highest for high monetary reward trials (*M* = 0.745, *SD* = 0.113), followed by both medium (*M* = 0.702, *SD* = 0.104) and low monetary reward trials (*M* = 0.707, *SD* = 0.115), *ps* < 0.001. However, in contrast to the liquid feedback condition, there was no difference between low and medium reward trials in the social feedback condition, *t*(73) = 0.65, *p* = 0.518, *d* = 0.05.

**FIGURE 4:**
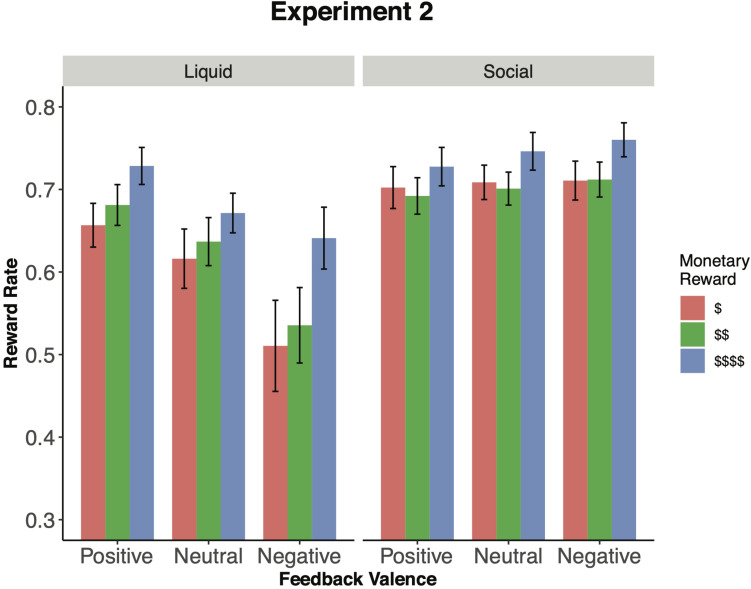
Incentive integration effects by experimental manipulation (Experiment 2). This figure shows reward rate by monetary reward level and feedback (i.e., liquid, social). Participants showed a main effect of monetary reward and feedback during both the social and liquid feedback sessions. However, the effect of feedback was not as strong for social relative to liquid feedback. Error bars represent 95% confidence intervals around the mean.

Next, we examined the effects of feedback valence. Although there was a main effect of feedback valence, *F*(2,146) = 12.34, *p* < 0.001, $\eta_{\text{G}}^{2}$ = 0.019, it was qualified by an interaction with incentive type, *F*(2,146) = 24.33, *p* < 0.001, $\eta_{\text{G}}^{2}$ = 0.035, which suggests that valence effects were distinct in the social relative to liquid feedback conditions. To decompose the feedback valence × incentive type interaction we examined the feedback valence effect in each incentive type (i.e., social, liquid) separately. There was an effect of valence for the liquid feedback condition, *F*(2,146) = 24.42, *p* = 0.009, $\eta_{\text{G}}^{2}$ = 0.027, that again replicated prior findings (Yee et al., 2016, 2019): reward rate was highest for positively valenced trials (Juice; *M* = 0.689, *SD* = 0.106) and lowest for negatively valenced trials [Saltwater; *M* = 0.562, *SD* = 0.212, *t*(73) = 5.42, *p* < 0.001, *d* = 0.71]; moreover, relative to neutral trials (Neutral; *M* = 0.641, *SD* = 0.154), positively valenced trials were significantly higher and negatively valenced trials were significantly lower in reward rate, *ps* ≤ 0.002 (Figure 4).

In contrast to the findings from Experiment 1, there was also an effect of valence in the social feedback condition, *F*(2,146) = 3.46, *p* = 0.034, $\eta_{\text{G}}^{2}$ = 0.004. However, in the social feedback condition the valence effect was *actually opposite to the predicted pattern*, and the effect observed in the liquid feedback condition: reward rate was highest for negative social feedback (*M* = 0.728, *SD* = 0.105), relative to positive social feedback [*M* = 0.707, *SD* = 0.113, *t*(73) = 2.88, *p* = 0.005, *d* = 0.18]. Further, there was no difference in reward rate between positive and neutral (*M* = 0.719, *SD* = 0.111), or neutral and negative feedback trials, *ps* ≥ 0.190. Feedback valence also interacted with monetary reward, *F*(4,292) = 3.48, *p* = 0.008, $\eta_{\text{G}}^{2}$ = 0.003. The 3-way interaction was not statistically significant, *F*(4,292) = 1.717, *p* = 0.146, $\eta_{\text{G}}^{2}$ = 0.002. These findings suggest that both primary incentive types (liquid, social) were able to modulate task performance. However, the social feedback effects were both qualitatively distinct and noticeably smaller in effect size from that observed in the liquid feedback condition (*d* = 0.18 social vs. *d* = 0.71 liquid), and moreover, opposite to what would be predicted from standard motivational valence account (i.e., negative > positive, rather than positive > negative).

### Discussion

In Experiment 2, we directly compared the effects of social incentives using static faces, relative to liquid incentives. Replicating Experiment 1 and prior work (Yee et al., 2016, 2019), in the liquid feedback condition we found effects of both monetary and liquid incentives on reward rate, which indicates that the liquid feedback manipulation was having a translatable effect to participants’ current motivational state. In contrast to the findings from Experiment 1, we also found an effect of social feedback on task performance; however, this effect was not as strong as the effect of liquid feedback and was also counterintuitive, in that it was *opposite to the expected pattern of results*, such that negative feedback elicited a slightly higher reward rate than positive social feedback. Taken together, these results provide strong confirmation of prior work (Yee et al., 2016, 2019), demonstrating that liquid incentives can modulate cognitive task performance through changes in experimentally-induced motivational states and highlight the lack of consistent integration effects for social feedback.

Further support for the distinct role of social feedback can be observed from the overall differences in task performance across both liquid and social conditions. Here, we observed higher overall reward rate (as well as higher accuracy; see Supplementary Material) in the social, relative to liquid, feedback condition. Although speculative, it is possible that the integration demands associated with the presence of two distinct incentives (monetary + liquid or social) increases the overall complexity of task demands (i.e., adding an additional cognitive load), which detracts from the ability to perform optimally in the heightened motivational context. If this is the case, then we would expect decreases in reward rate from positive to neutral to negative feedback valence scaled with the attainable monetary rewards. Though this is the exact pattern of results we observe in the liquid feedback condition, these effects are weaker in the social feedback condition. Thus, the results from the social feedback condition could indicate performance operating at ceiling levels, which could occur if the two incentives are actually not being directly integrated together in a valence-dependent manner, as appears to occur in the liquid feedback condition. Such an explanation could also potentially account for the qualitatively distinct valence effects observed in the social feedback condition. In particular, it is possible that such effects reflect an arousal rather than a true valence and integration effect, which is plausible given prior findings suggesting that negatively valenced facial expressions tend to be more arousing than positively valenced ones (Duval et al., 2013). Of course, these hypotheses would need to be supported by future work, which should benchmark liquid and social feedback conditions against incentive conditions that do not involve integration demands (e.g., a monetary incentive only condition).

## General Discussion

Across two experiments we found strong evidence supporting the role of liquid rewards as a robust motivationally-valenced incentive that can be integrated with monetary incentives to modulate cognitive task performance. This work extends our prior studies with the liquid feedback incentive integration paradigm (Yee et al., 2016, 2019) in two ways. First, we found that in addition to its motivational impact on behavioral performance in a challenging cognitive task, liquid feedback also influenced participants’ self-reported affect in valence-specific ways, with appetitive liquid feedback increasing self-reported positive affect, and aversive liquid feedback increasing self-reported negative affect. Second, and most critically, the two studies converged in demonstrating that primary liquid incentives had a reliably stronger impact on task performance than did social incentives. This last point is critical, in that liquid and social incentives were directly compared with a paradigm specifically optimized to assess the motivational influence of non-monetary incentives, with a design that enabled the effect of these incentives to be measured in an incidental fashion, that is, in a manner less susceptible to demand characteristics or other confounding factors than other experimental approaches used in the literature.

Surprisingly, the findings with respect to social incentives across the two studies were counter to our initial predictions. In particular, we observed weak or null effects of social feedback on task performance in both studies. Moreover, we found preliminary evidence for a dissociation between affective and motivational responses to the social feedback, such that social incentives appear to impact participants’ self-reported affective state, but not their motivation. Taken together, these results provide preliminary evidence of the dissociable of effects of primary reward type (i.e., liquid, social) in motivating cognitive behavior, whereby liquid incentives appear to induce both strong affective and motivational effects, whereas social incentives have a limited impact on behavior.

Despite the null or inconsistent effects of social feedback found in this study, it is important to consider the possibility that there are real motivational consequences of social incentives, but that our methods were not sufficiently sensitive to them, given the potential limitations associated with our current experimental paradigm and/or the social stimuli we used. For example, an alternative and potentially promising approach would be to test our social stimuli with a simpler task, rather than the highly challenging cued-task switching paradigm used in the current study, such as the Monetary Incentive Delay (MID) task (Knutson et al., 2003). Indeed, work using the MID has shown greater activity modulation in the right nucleus accumbens to social reward (smiling faces of differing intensities) relative to monetary reward, but the opposite pattern for behavioral results (i.e., higher hit rate for monetary relative to social incentives; Rademacher et al., 2014). Likewise, studies using simple approach or avoidance movements (i.e., arm extension or flexion) have found valenced effects to social stimuli (Nikitin and Freund, 2019). Thus, it is possible that we would have obtained a different pattern of results, if we had employed simpler tasks and/or behavioral response metrics.

Another possible interpretation of our findings is that even though the social stimuli were selected to increase ecological validity (e.g., using short videos clips and images of actual faces), they may have actually been ineffective in modulating motivational state. The stimuli, though selected to be meaningful and motivating (in both positive and negative directions), may have seemed artificial to the participants, and could have been easily ignored. Indeed, it is possible that participants switched strategies for the social feedback condition, relative to liquid feedback, and selectively attended to the monetary reward cues (e.g., $$, $$$$) to guide performance, effectively ignoring the messages provided by the feedback stimuli. If attention was more strongly directed to the monetary incentive cues in the social feedback condition, then we would have predicted stronger monetary effects relative to the liquid feedback condition. In fact, we observed the opposite pattern, in that monetary reward effects were also weaker in the social feedback conditions relative to liquid feedback.

Further, the stimuli may not have been personally meaningful, in that the feedback was presented by unfamiliar actors (section “Experiment 1”) or unfamiliar faces (section “Experiment 2”), rather than by individuals known or influential to the participants. For example, presenting participants with social feedback provided by known and influential figures, such as friends or family members, could have been more effective, and more reflective of these experiences in daily life contexts. Indeed, recent work has shown that social closeness, as indexed by the degree to which participants rated how much they liked the person giving them social feedback, modulated activity in the ventral striatum in response to feedback, and also increased favorable impressions of the person after receiving positive social feedback from them (Hughes et al., 2018). Studies from daily life also support the idea that there is strong affective coupling between an individual and their close social partners (Mejía and Hooker, 2015), which could suggest that messages delivered by close others would have a stronger impact on affect and motivation than unknown actors. Further, other work also suggests that diverse incentive types (e.g., money, food, social) have similar motivational effects when they are equated in subjective value (Lehner et al., 2017). Thus, it is possible that our social stimuli might exhibit equivalent motivational effects to the liquid stimuli, if we were to carefully match each participant’s incentive value across liquid and social domains prior to performing the incentive integration paradigm.

Nevertheless, it is worth noting that these critiques apply to almost all of the existing laboratory studies that have examined processes related to social reward and motivation. Most of these studies use fairly impoverished stimuli, like emoticons or sentences relaying social information, which also could be construed as being artificial and would seem to be even more easily ignored. Despite these potential shortcomings, prior studies utilizing these relatively impoverished stimuli have shown significant responses in the neural regions associated with reward processing (i.e., striatum, vmPFC), such as when the social stimuli are images of static faces (Cloutier et al., 2008; Spreckelmeyer et al., 2009), or feedback messages consisting only of written sentences or even just numerical ratings (Hughes et al., 2018; Izuma et al., 2008; Korn et al., 2012). Thus, it is possible that the social incentives utilized in this study may have elicited reward related neural activity.

A related concern associated with the prior literature is that there is little evidence of robust brain-behavior relationships in studies using social rewards to identify reward-related neural activity. Indeed, recent work has found differential patterns of behavior across incentive modalities, in that performance costs were only observed for social stimuli, while performance benefits were only observed for monetary rewards on a cognitive control task, despite shared activation patterns and magnitude of response across value-encoding regions of the brain to both monetary and social incentives (Park et al., 2018). These findings suggest that social incentives can engage the same brain regions that support the processing of a diverse range of rewarding stimuli to motivate behavior, even when the social stimuli aren’t very life-like or particularly social in nature. Yet there is little support for the assumption that these types of social stimuli will motivate changes or enhancements in goal-directed behavior. Such findings highlight the distinct possibility that social motivational variables are actually less effective, or at least less consistent, than other types of incentive modalities in motivating behavior, even when they are accompanied by equivalent neural responses to reward.

In summary, our results do show that provided with social incentives, participants are able to improve their performance on a challenging cognitive task relative to a non-rewarded baseline condition. Nevertheless, despite popular theorizing that social incentives are potentially more motivating to humans than other primary incentives, our findings stand in contrast to this claim. In our incentive integration cued task-switching paradigm, social incentives show clearly weaker and qualitatively distinct influences on motivated cognitive control and associated task performance than do liquid incentives. Future work is needed to characterize the role that social incentives play in motivating goal-directed behaviors, and how, or if, different components of reward processing (i.e., wanting, liking) to social stimuli contribute to such behaviors. This type of research is essential for understanding how diverse incentive types are used to guide decision-making processes in everyday life and motivate the successful mobilization of cognitive control.

## Data Availability Statement

The datasets presented in this study can be found in online repositories. The names of the repository/repositories and accession number(s) can be found in the article/Supplementary Material.

## Ethics Statement

The studies involving human participants were reviewed and approved by Washington University Human Research Protections Office. The patients/participants provided their written informed consent to participate in this study.

## Author Contributions

JC, DY, HH, RT, and TB designed the research. JC, AN, and KS performed the research. JC, DY, HH, AN, KS, and TB analyzed the data. JC wrote the first draft of the manuscript. All authors edited the manuscript.

## Conflict of Interest

The authors declare that the research was conducted in the absence of any commercial or financial relationships that could be construed as a potential conflict of interest.

## Footnotes

1 https://peplab.ucdavis.edu/videos.php

2 https://danlab7.wixsite.com/nimstim
