# Supplementary material for: Dissociable Effects of Monetary, Liquid, and Social Incentives on Motivation and Cognitive Control
Source: Front Psychol. 2020 Sep 9;11:2212. doi: 10.3389/fpsyg.2020.02212 (PMC7509070; doi:10.3389/fpsyg.2020.02212)
Supplement: Supplementary file 1 [file Data_Sheet_1.PDF]

Supplemental Material

Dissociable effects of monetary, liquid, and social incentives on motivation and cognitive control

Experiment I-Within-Subject Manipulation of both social (dynamic stim) and liquid feedback

S1. Summary Table of Reward Rate, Accuracy, Error Rate, and RT

Table 1: Task Performance Variables

		Liquid								Social							
		Reward Rate		Accuracy		Error Rate		Reaction Time		Reward Rate		Accuracy		Error Rate		Reaction Time	
Feedback Valence	Monetary Reward	Mean	SD	Mean	SD	Mean	SD	Mean	SD	Mean	SD	Mean	SD	Mean	SD	Mean	SD
**Negative**																	
Negative	Low	0.560	0.183	0.774	0.120	0.045	0.037	516.466	105.781	0.657	0.122	0.837	0.100	0.037	0.046	485.989	50.971
Negative	Medium	0.556	0.185	0.796	0.139	0.049	0.050	525.400	96.434	0.669	0.092	0.860	0.072	0.038	0.033	488.338	71.513
Negative	High	0.626	0.173	0.810	0.145	0.041	0.051	477.587	76.576	0.676	0.102	0.835	0.057	0.025	0.039	471.212	57.764
**Neutral**																	
Neutral	Low	0.650	0.123	0.839	0.065	0.046	0.041	483.647	72.863	0.622	0.138	0.829	0.088	0.040	0.038	494.031	58.394
Neutral	Medium	0.609	0.138	0.829	0.079	0.040	0.035	496.988	81.683	0.649	0.124	0.869	0.069	0.045	0.038	499.539	56.108
Neutral	High	0.693	0.111	0.831	0.087	0.028	0.038	458.348	58.663	0.688	0.099	0.861	0.051	0.022	0.032	471.298	46.767
**Positive**																	
Positive	Low	0.660	0.120	0.818	0.092	0.032	0.031	461.462	67.753	0.644	0.125	0.849	0.087	0.045	0.038	491.220	51.494
Positive	Medium	0.642	0.102	0.815	0.080	0.048	0.031	471.622	71.874	0.628	0.128	0.853	0.102	0.046	0.062	496.927	51.733
Positive	High	0.686	0.105	0.835	0.101	0.024	0.029	453.610	56.001	0.707	0.106	0.870	0.073	0.026	0.033	468.047	44.320

Comparing Reward Rate Across both Experimental Sessions

S2. Reward Rate by Incentive Type

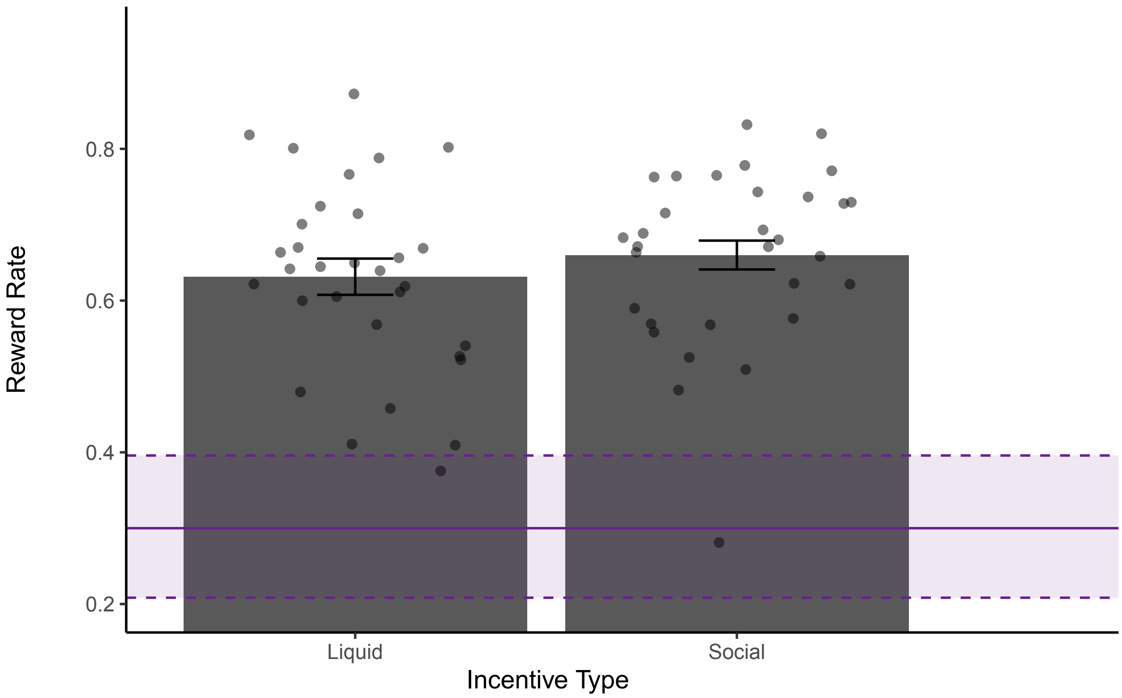

S3. Reward Rate by Incentive Type and Session

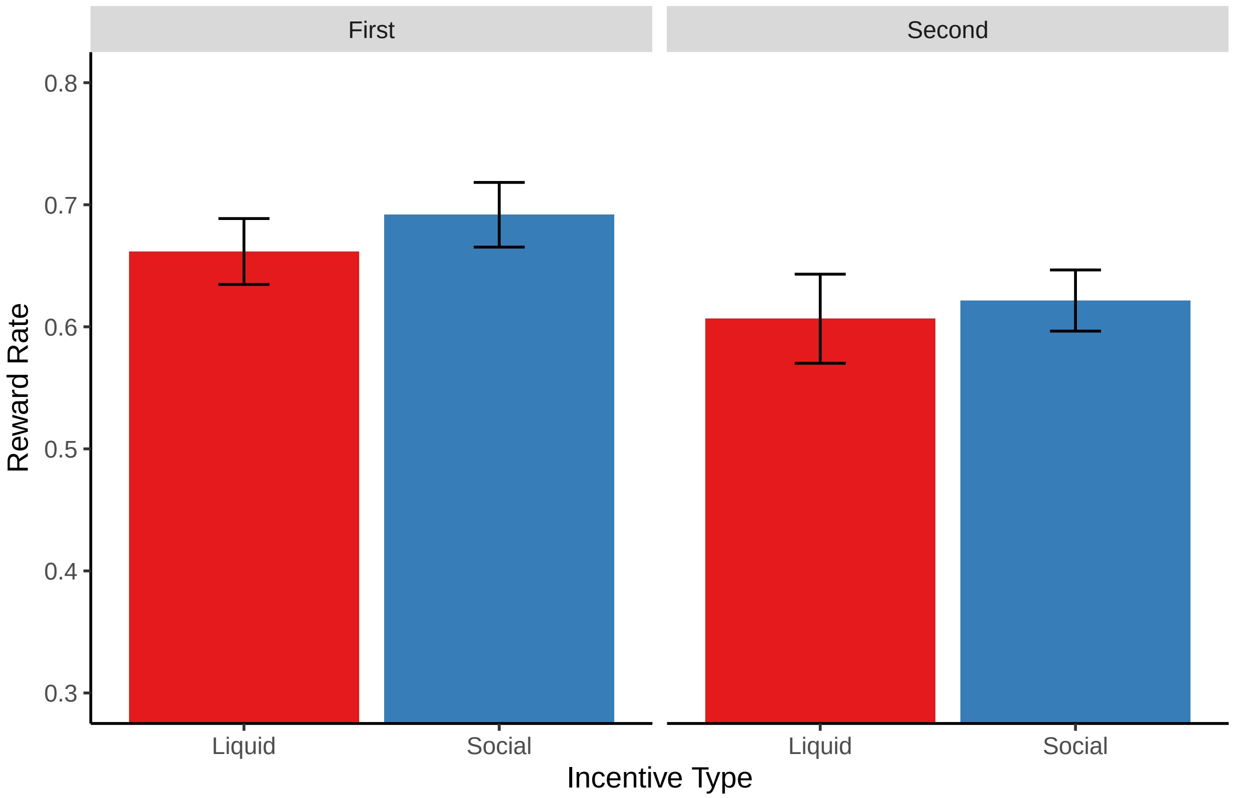

S4. Accuracy Across Incentive Type and Session

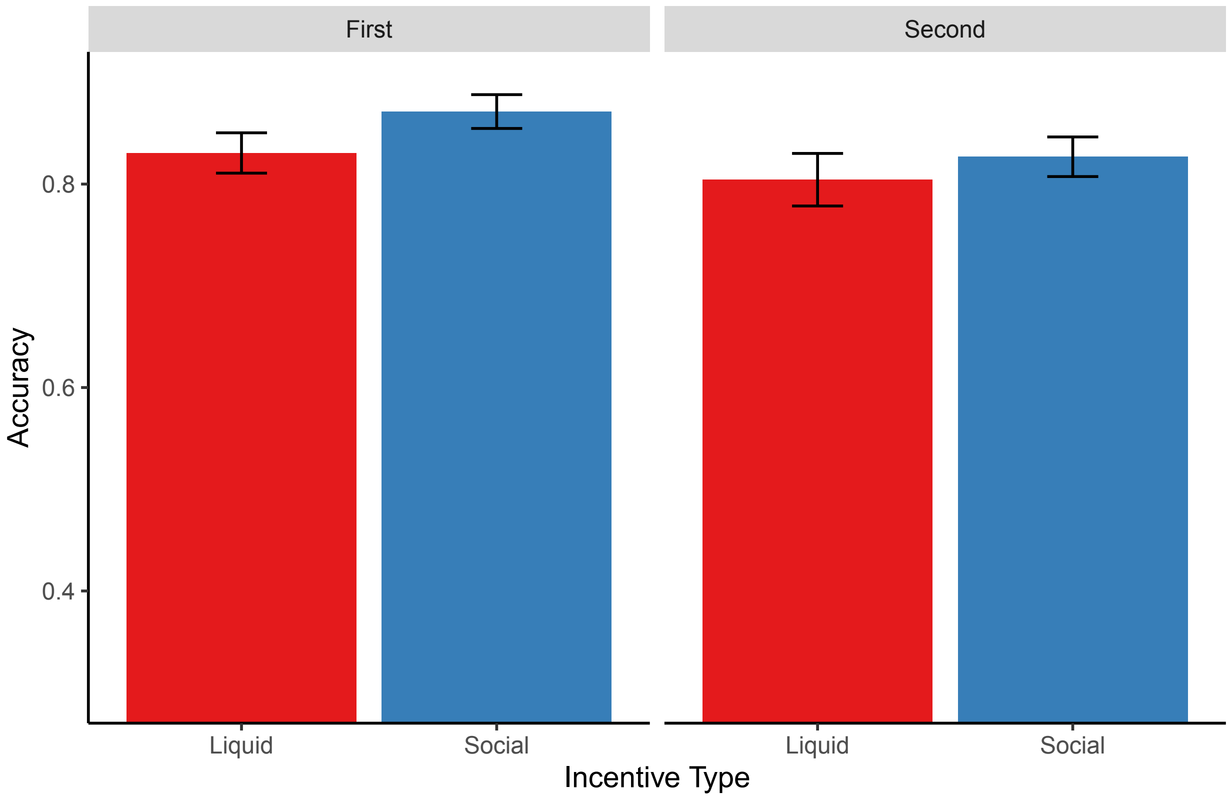

S5. RT Across Incentive Type and Session

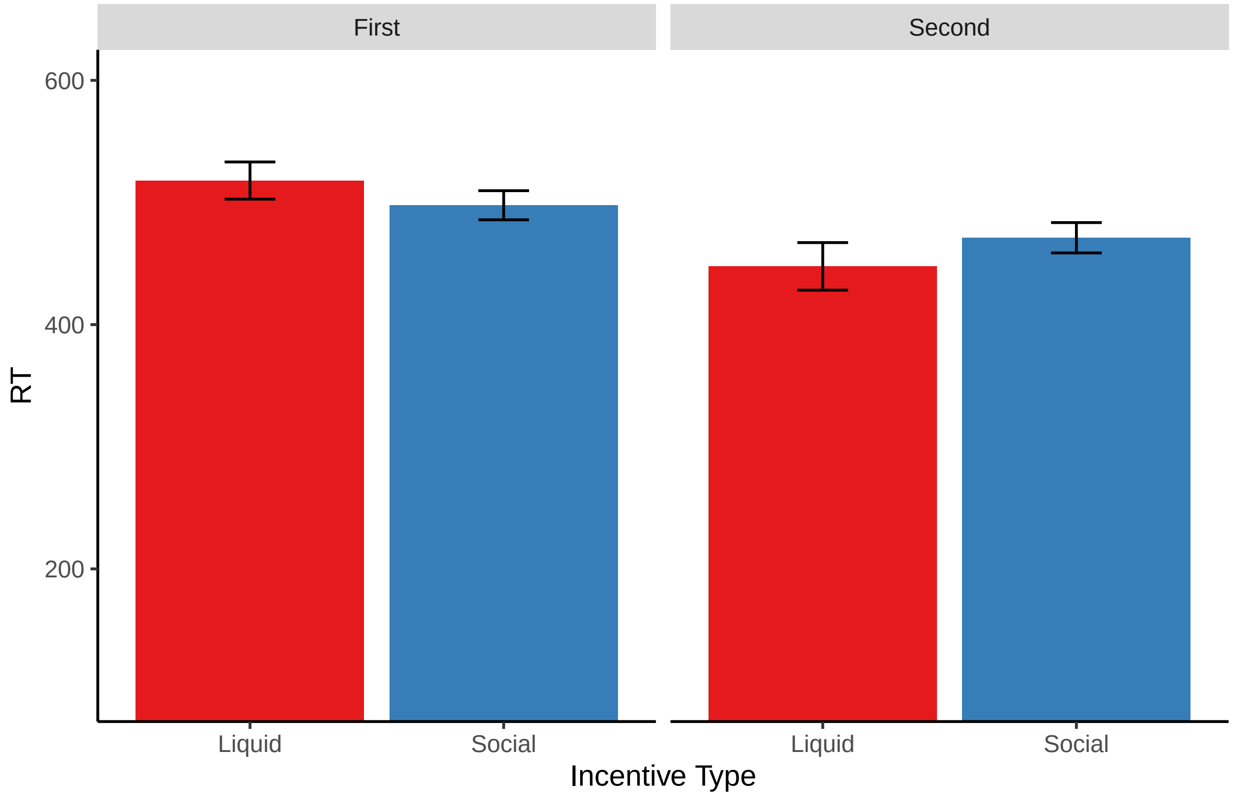

S6. Affect Ratings Summary

Feeling	Experiment	meanRating
Alert	Liquid Feedback	3.333333
Alert	Social Feedback	2.956989
Ashamed	Liquid Feedback	1.376344
Ashamed	Social Feedback	1.451613
Content	Liquid Feedback	2.731183
Content	Social Feedback	2.516129
Determined	Liquid Feedback	3.236559
Determined	Social Feedback	3.021505
Excited	Liquid Feedback	2.279570
Excited	Social Feedback	1.935484
Fatigued	Liquid Feedback	2.440860
Fatigued	Social Feedback	2.892473
Inspired	Liquid Feedback	1.967742
Inspired	Social Feedback	1.903226
Irritable	Liquid Feedback	2.053763
Irritable	Social Feedback	1.903226
Stressed	Liquid Feedback	2.021505
Stressed	Social Feedback	2.043011
Upset	Liquid Feedback	1.784946
Upset	Social Feedback	1.677419

S7. Self-Reported Motivation Ratings

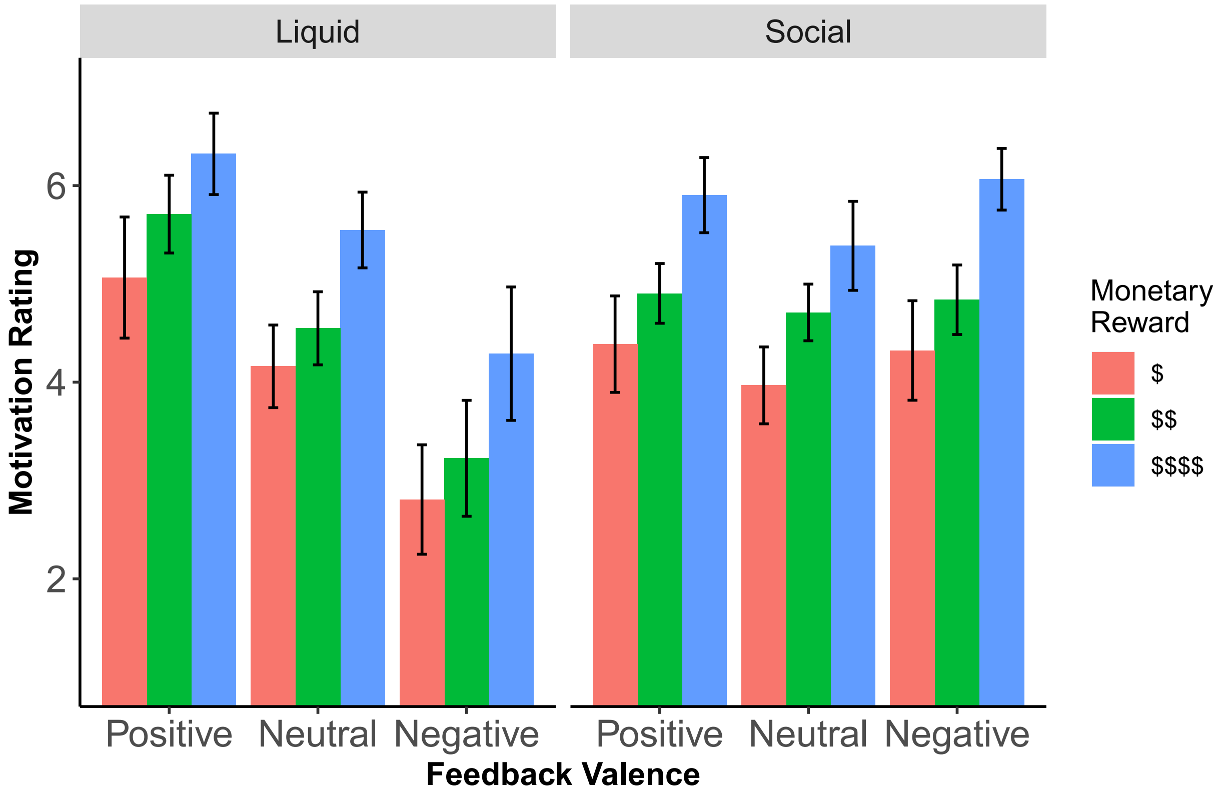

Post-Task Motivation Ratings

Feedback Valence	Monetary Reward	Liquid		Social	
		Mean	SD	Mean	SD
**Negative**					
	Low	2.806	1.518	4.323	1.380
	Medium	3.226	1.608	4.839	0.965
	High	4.290	1.851	6.065	0.855
**Neutral**					
	Low	4.161	1.146	3.968	1.066
	Medium	4.548	1.014	4.710	0.785
	High	5.548	1.050	5.387	1.234
**Positive**					
	Low	5.065	1.680	4.387	1.338
	Medium	5.710	1.078	4.903	0.829
	High	6.323	1.129	5.903	1.043

S8. Self-Reported Liking Ratings

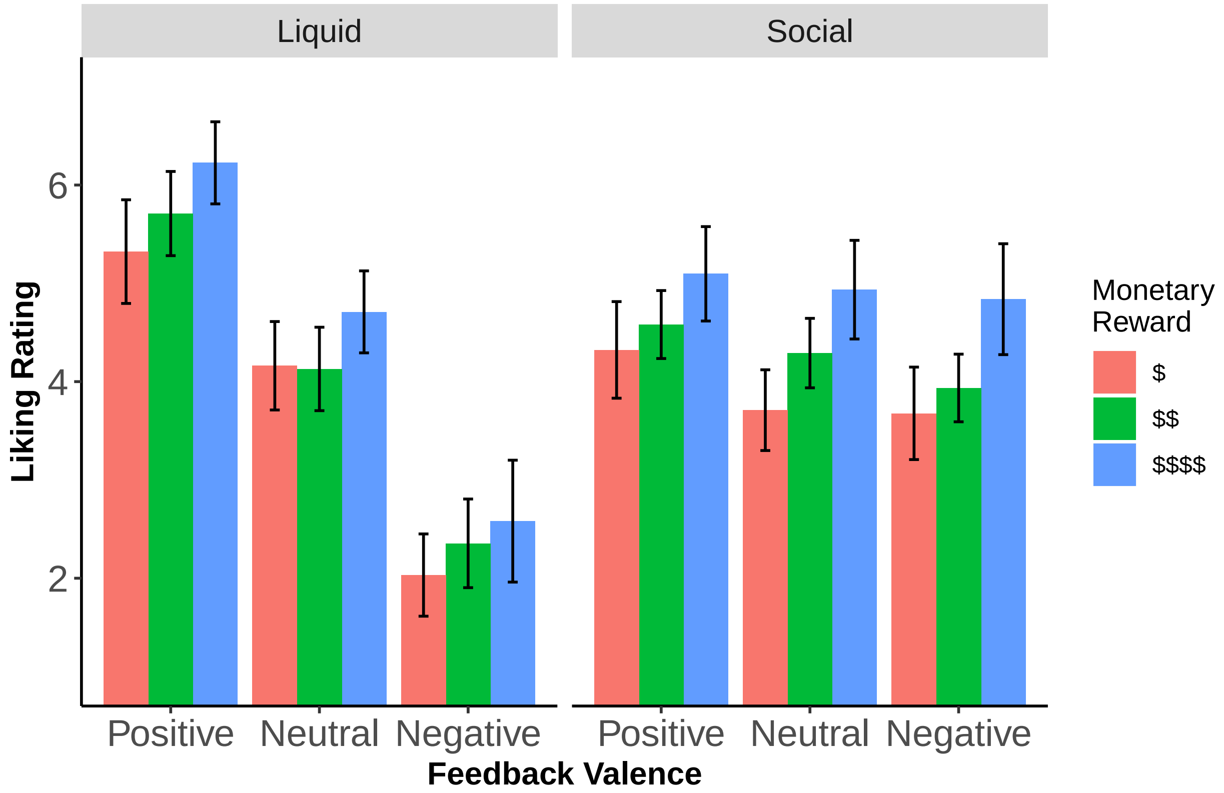

Post-Task Liking Ratings

		Liquid		Social	
Feedback Valence	Monetary Reward	Mean	SD	Mean	SD
**Negative**					
	Low	2.032	1.141	3.677	1.283
	Medium	2.355	1.229	3.935	0.938
	High	2.581	1.690	4.839	1.538
**Neutral**					
	Low	4.161	1.226	3.710	1.119
	Medium	4.129	1.157	4.290	0.963
	High	4.710	1.137	4.935	1.368
**Positive**					
	Low	5.323	1.437	4.323	1.340
	Medium	5.710	1.167	4.581	0.943
	High	6.226	1.139	5.097	1.309

S9. Individual Difference Questionnaire Summary

BIS/BAS Means

BAS Reward	BAS Reward (sd)	BAS Fun	BAS Fun (sd)	BAS Drive	BAS Drive (sd)	BIS
22.41935	2.655487	14.96774	3.737057	22.41935	3.180924	25.3871

DARS Means

DARS Hobby	DARS Hobby (sd)	DARS Food	DARS Food (sd)	DARS Social	DARS Social (sd)	DARS Sensory	DARS Sensory (sd)
15.29032	1.465004	14.06452	2.06455	13.16129	2.23751	15.74194	4.289271

Experiment II - Within-Subject Manipulation of both social (static stim) and liquid feedback

S10. Summary Table of Reward Rate, Accuracy, Error Rate, and RT (Experiment II)

Task Performance Variables

		Liquid								Social							
		Reward Rate		Accuracy		Error Rate		Reaction Time		Reward Rate		Accuracy		Error Rate		Reaction Time	
Feedback Valence	Monetary Reward	Mean	SD	Mean	SD	Mean	SD	Mean	SD	Mean	SD	Mean	SD	Mean	SD	Mean	SD
**Negative**																	
Negative	Low	0.511	0.238	0.690	0.242	0.053	0.078	613.402	138.251	0.711	0.102	0.865	0.090	0.030	0.034	548.123	46.697
Negative	Medium	0.535	0.197	0.735	0.208	0.043	0.055	611.458	105.986	0.712	0.091	0.877	0.071	0.028	0.035	551.252	49.291
Negative	High	0.641	0.162	0.797	0.136	0.037	0.039	567.344	95.681	0.760	0.089	0.883	0.076	0.019	0.027	531.422	44.855
**Neutral**																	
Neutral	Low	0.616	0.155	0.811	0.141	0.046	0.049	576.903	73.455	0.709	0.090	0.869	0.076	0.035	0.036	553.126	44.661
Neutral	Medium	0.637	0.126	0.839	0.102	0.035	0.040	576.463	72.821	0.701	0.086	0.875	0.078	0.030	0.036	554.327	41.506
Neutral	High	0.671	0.104	0.830	0.086	0.041	0.045	545.730	51.642	0.746	0.099	0.878	0.084	0.023	0.031	534.400	40.584
**Positive**																	
Positive	Low	0.657	0.114	0.840	0.074	0.043	0.043	560.670	53.857	0.702	0.110	0.879	0.086	0.024	0.036	557.400	49.939
Positive	Medium	0.681	0.107	0.861	0.072	0.038	0.036	561.586	58.146	0.692	0.095	0.866	0.088	0.030	0.031	558.283	50.756
Positive	High	0.728	0.097	0.877	0.070	0.022	0.027	539.483	52.450	0.728	0.101	0.875	0.077	0.027	0.038	540.874	49.301

S11. Reward Rate by Incentive Type (Experiment II)

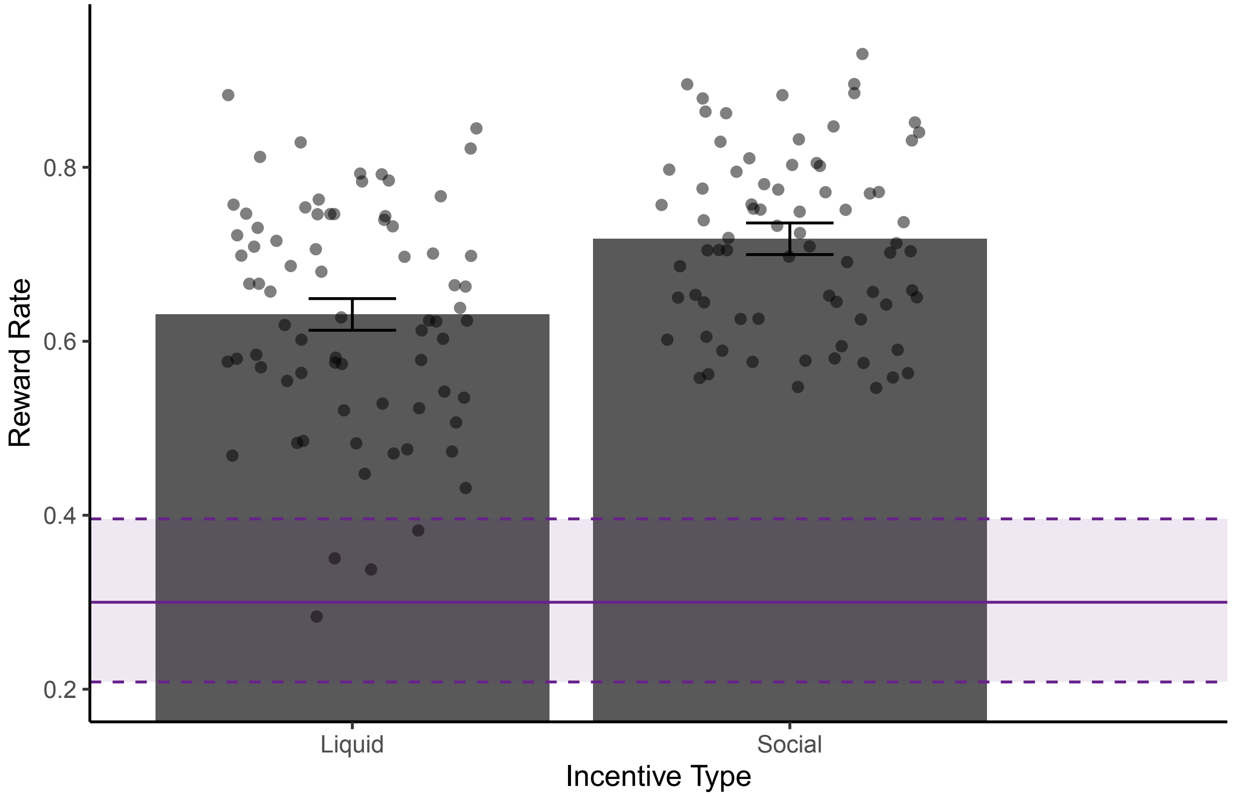

S12. Self-Reported Motivation Ratings (Experiment II)

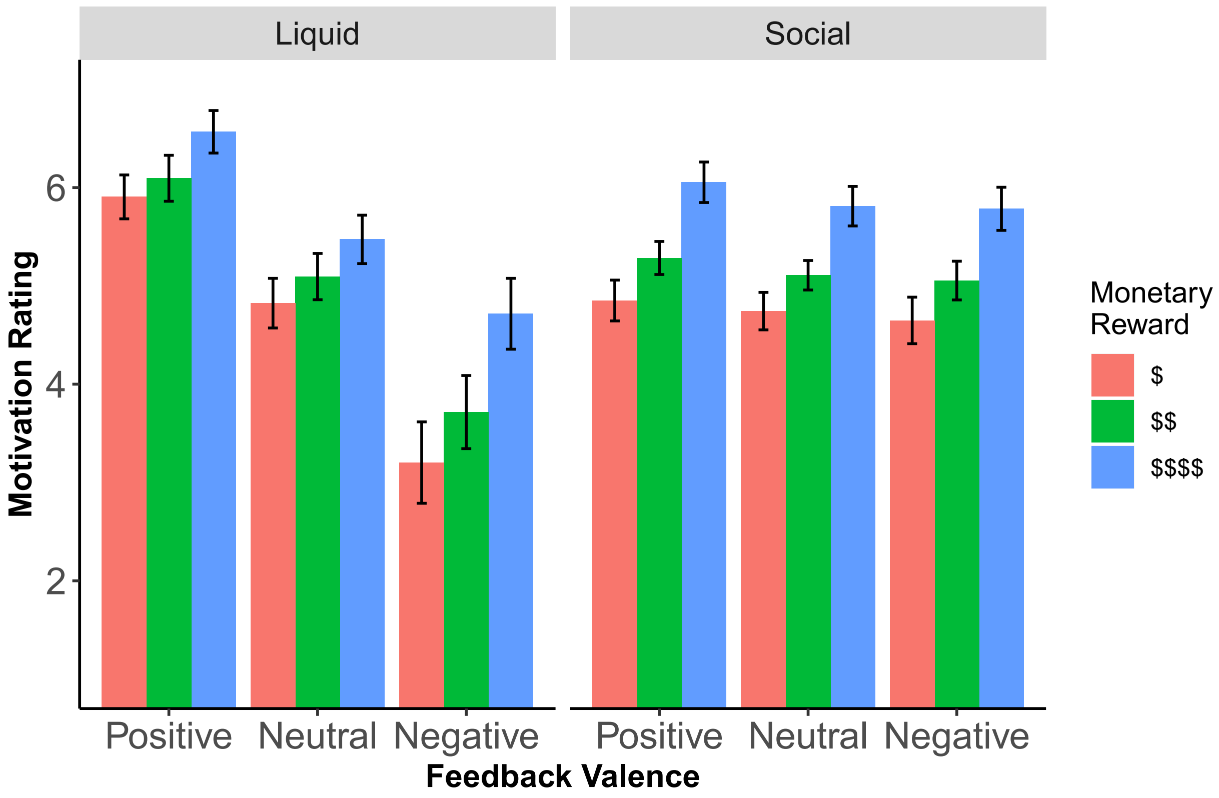

Post-Task Motivation Ratings

		Liquid		Social	
Feedback Valence	Monetary Reward	Mean	SD	Mean	SD
**Negative**					
	Low	3.203	1.788	4.649	1.021
	Medium	3.716	1.606	5.054	0.849
	High	4.716	1.556	5.784	0.946
**Neutral**					
	Low	4.824	1.090	4.743	0.822
	Medium	5.095	1.015	5.108	0.648
	High	5.473	1.062	5.811	0.867
**Positive**					
	Low	5.905	0.964	4.851	0.896
	Medium	6.095	1.009	5.284	0.726
	High	6.568	0.934	6.054	0.889

S13. Self-Reported Liking Ratings (Experiment II)

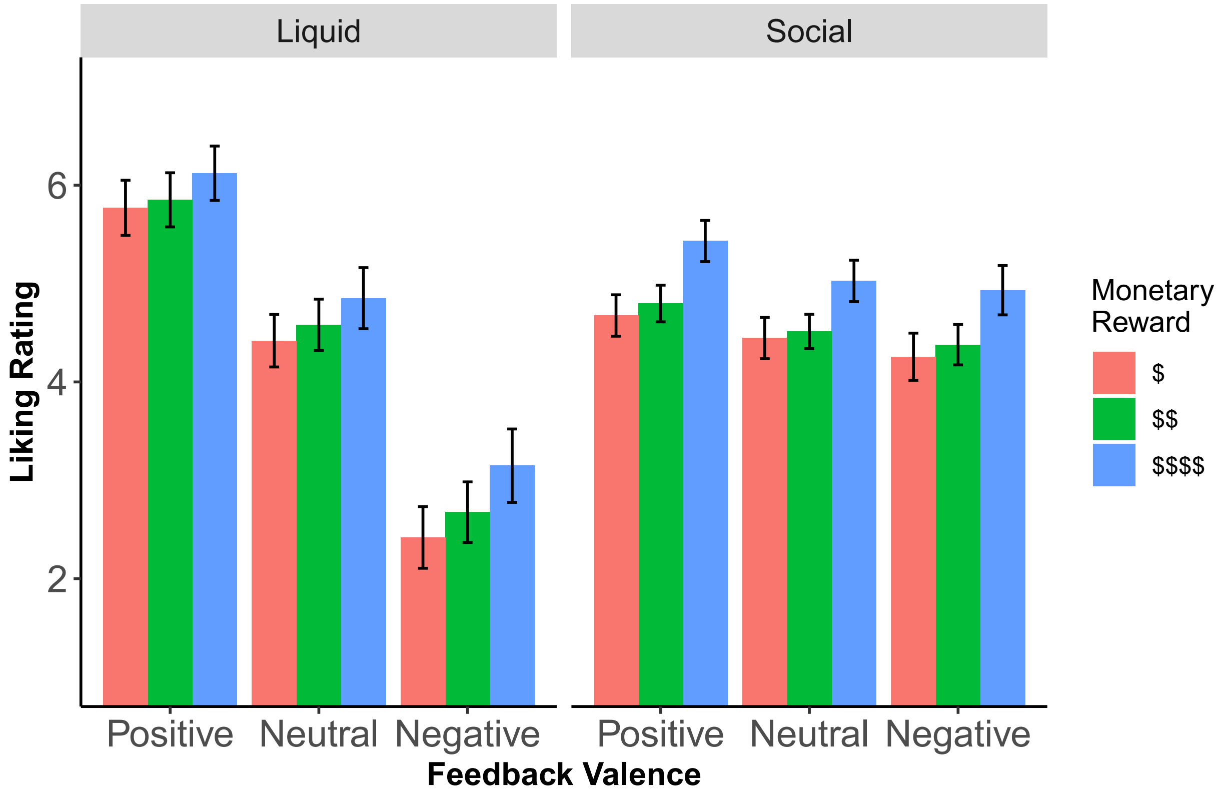

Post-Task Liking Ratings

		Liquid		Social	
Feedback Valence	Monetary Reward	Mean	SD	Mean	SD
**Negative**					
	Low	2.419	1.351	4.257	1.034
	Medium	2.676	1.330	4.378	0.887
	High	3.149	1.610	4.932	1.082
**Neutral**					
	Low	4.419	1.152	4.446	0.906
	Medium	4.581	1.125	4.514	0.754
	High	4.851	1.340	5.027	0.910
**Positive**					
	Low	5.770	1.209	4.676	0.908
	Medium	5.851	1.188	4.797	0.806
	High	6.122	1.193	5.432	0.904

S14. Individual Difference Questionnaire Summary (Experiment II)

BIS/BAS Means

BAS Reward	BAS Reward (sd)	BAS Fun	BAS Fun (sd)	BAS Drive	BAS Drive (sd)	BIS
17.54054	1.980654	12.09459	2.439228	17.54054	2.417732	21.16216

DARS Means

DARS Hobby	DARS Hobby (sd)	DARS Food	DARS Food (sd)	DARS Social	DARS Social (sd)	DARS Sensory	DARS Sensory (sd)
14.75676	2.298366	12.54054	3.043921	13.31081	2.363685	16.55405	3.875253

MASQ Means

mean__MASQ__anhed	sd__MASQ__anhed	mean__MASQ__anhed8	sd__MASQ__anhed8
52.12162	12.75368	15.37838	4.764891
